# ALFA: annotation landscape for aligned reads

**DOI:** 10.1186/s12864-019-5624-2

**Published:** 2019-03-29

**Authors:** Mathieu Bahin, Benoit F. Noël, Valentine Murigneux, Charles Bernard, Leila Bastianelli, Hervé Le Hir, Alice Lebreton, Auguste Genovesio

**Affiliations:** 1 Computational Biology and Bioinformatics group,, Institut de biologie de l’ENS (IBENS), Département de biologie, École normale supérieure, CNRS, INSERM, PSL University, 75005, Paris, France; 2 Bacterial Infection & RNA Destiny group, Institut de biologie de l’ENS (IBENS), Département de biologie, École normale supérieure, CNRS, INSERM, PSL University, 75005, Paris, France; 3 Expression of eukaryotic messenger RNAs group, Institut de biologie de l’ENS (IBENS), Département de biologie, École normale supérieure, CNRS, INSERM, PSL University, 75005 Paris, France; 4 INRA, IBENS, 75005 Paris, France

**Keywords:** NGS, Quality control, Post mapping, Universal, Tool

## Abstract

**Background:**

The last 10 years have seen the rise of countless functional genomics studies based on Next-Generation Sequencing (NGS). In the vast majority of cases, whatever the species, whatever the experiment, the two first steps of data analysis consist of a quality control of the raw reads followed by a mapping of those reads to a reference genome/transcriptome. Subsequent steps then depend on the type of study that is being made. While some tools have been proposed for investigating data quality after the mapping step, there is no commonly adopted framework that would be easy to use and broadly applicable to any NGS data type.

**Results:**

We present ALFA, a simple but universal tool that can be used after the mapping step on any kind of NGS experiment data for any organism with available genomic annotations. In a single command line, ALFA can compute and display distribution of reads by categories (exon, intron, UTR, etc.) and biotypes (protein coding, miRNA, etc.) for a given aligned dataset with nucleotide precision. We present applications of ALFA to Ribo-Seq and RNA-Seq on *Homo sapiens*, CLIP-Seq on *Mus musculus*, RNA-Seq on *Saccharomyces cerevisiae*, Bisulfite sequencing on *Arabidopsis thaliana* and ChIP-Seq on *Caenorhabditis elegans*.

**Conclusions:**

We show that ALFA provides a powerful and broadly applicable approach for post mapping quality control and to produce a global overview using common or dedicated annotations. It is made available to the community as an easy to install command line tool and from the Galaxy Tool Shed.

## Background

Software programs such as FastQC [1] have become routine to get information about raw high-throughput sequenced material. Most of the time, this first analysis step is followed by a second step where the reads are mapped to a reference genome using a sequence aligner such as STAR [2]. However, the subsequent steps are often very specific to the type of NGS experiment that is being run. For instance, in ChIP-Seq [3] or CLIP-Seq [4] experiments, peaks will need to be detected prior to further processing; in RNA-Seq, a differential analysis will often be performed on aligned reads; in BS-Seq experiments, a dedicated analysis of sequence will be applied in order to obtain a methylation ratio per nucleotide prior to further processing. To sum up, only two steps are common to a large majority of NGS data analysis pipelines: sequencing quality control and mapping. The subsequent steps are most often specific to the considered application.

Dedicated categorization of reads for specific kinds of NGS data were employed in past studies performing RNA-Seq [5], Ribosome profiling [6], ChIP-seq [7] or miR-seq [8]. However, they were not designed to be broadly applicable. That is, none of these dedicated methods would be suitable for the analysis of another type of NGS data. For instance, a peak calling was a prerequisite to ChIP-Seq data categorization in [9] which would be meaningless for an RNA-Seq experiment. Alternatively and with some coding effort, programs such as BEDTools [10], BEDOPS [11], featureCounts or Bam2x [12] can be combined to intersect positions with feature intervals and produce read feature distributions (see Table 1 for a comparison). However, combining those is not straightforward and the overall process is often largely sub-optimal [13]. To the best of our knowledge, there is no available tool that provides a broadly applicable and fast quantitative overview of several samples with nucleotide precision.

**Table 1:** Comparison of available tools with functionalities possibly overlapping with ALFA. This table shows that none of them offers all capabilities provided by ALFA

	BEDOPS [11]	BEDTools [10]	featureCounts [25]	CAP-miRSeq [8]	HOMER [9]	RSeqQC [26]	bam2x [12]	CEAS [7]	ALFA
One line call				✔⃞	✔⃞	✔⃞	✔⃞	✔⃞	✔⃞
Multiple samples			✔⃞	✔⃞					✔⃞
Nucleotide resolution	✔⃞	✔⃞							✔⃞
Hierarchical priorities			✔⃞		✔⃞	✔⃞	✔⃞	✔⃞	✔⃞
Enrichment score						✔⃞		✔⃞	✔⃞
Categories	✔⃞	✔⃞	✔⃞		✔⃞	✔⃞	✔⃞	✔⃞	✔⃞
Biotypes	✔⃞	✔⃞	✔⃞	✔⃞					✔⃞
Graphical output				✔⃞			✔⃞	✔⃞	✔⃞
Any NGS data	✔⃞	✔⃞	✔⃞			✔⃞	✔⃞		✔⃞

Here, we introduce ALFA (Annotation Lanscape For Aligned reads), a simple and broadly applicable tool that produces a global overview of the distribution of mapped reads, both in terms of genomic categories (stop codon, 5′-UTR, CDS, intergenic, etc.) and biotypes (protein coding, miRNA, ncRNA, etc.) with nucleotide precision. ALFA turned out to be a very useful systematic post-mapping quality control tool for a broad range of NGS applications we have been dealing with. We describe here the results obtained with ALFA using datasets produced by several types of NGS experiments on various model organisms. Even though its primary purpose was to offer a quality control step right after mapping, we show that ALFA occasionally provided a first global functional insight prior to a more dedicated analysis.

## Implementation

Common genomic databases (e.g. Ensembl) mostly propose two types of functional annotations: genomic categories (5 ´-UTR, CDS, intron, intergenic region, etc.) and gene biotypes (protein-coding, miRNA, IG-gene, etc.). An annotation file with both the category and biotype information is required to run ALFA. In order to compute the proportions of reads from a given NGS dataset falling into these annotated regions, ALFA proceeds in three steps. In a first step, ALFA generates reusable index files from genomic annotations. In a second step, ALFA computes the actual nucleotide count per category from a given set of NGS samples using those index files. Finally, ALFA can display bar plots of the raw and normalized distributions. Importantly, the three steps described in detail below can be processed at once with a single command line call.

### Creation of the index files

ALFA first processes a GTF annotation file and converts it to two index files. This step needs to be performed only once for a given annotation file. Its aim is to transform the GTF file content, which is an unordered and overlapping set of associations between intervals and category-biotype pairs, into a list of category-biotype pairs indexed by genomic coordinates. For instance, in a GTF file, a nucleotide could be associated to an exon interval but also to at least one transcript and one gene interval. This hierarchy can be deeper for the most precise features (UTR, CDS, start codon, etc.). Consequently, in a GTF file most genomic coordinates appear several times and in an unordered fashion. In order to suppress this redundancy, this indexing step splits intervals so as to remove overlaps and so that each interval matches a list of category-biotype pairs. It proceeds as follows: for each row of the GTF file, if the category-biotype pair for the considered interval does not overlap with any yet processed intervals, it is created. If a nucleotide sequence belongs to several overlapping features at the same hierarchical level, it is considered as ambiguous by default and discarded from further processing. Optionally, ALFA can equally split the considered counts between the overlapping category-biotype pairs. At the end of the process, the index is made of non overlapping intervals annotated with one or more category-biotype pairs. In this way, all category-biotypes of any given genomic coordinate can be retrieved at once. Furthermore, in order to preserve the DNA strand information, two index files are created. The first one is a stranded index, which contains the features for the sense and for the antisense strands separately. In stranded mode, a nucleotide mapping to an unannotated region on one strand facing an annotated region on the opposite strand will be counted in the category “opposite strand”. The second one is an unstranded index containing all annotations from both strands. Concretely, the systematic production of those two files enables a flexible choice of strandness when processing sample files at the next step using the strand parameter.

### Processing of the sample files

The processing of a set of mapped reads samples in BAM format is performed in two steps. First the coverage per genomic position is obtained for each sample using Bedtools genomecov [10] which produces a bedgraph file per sample. The bedgraph format is a non overlapping list of genomic intervals with counts. Following this, each bedgraph file is intersected with the index previously computed from the GTF file (either the stranded or the unstranded version, according to the strand parameter chosen by the user). This intersection consists in aggregating all counts falling into all intervals of each category-biotype pair. Concretely, it is obtained in the following way: each genomic interval defined in the bedgraph files is split into as many genomic intervals it covers in the index file and the list of category-biotype associations found in those intervals are incremented by the corresponding fraction of the count. As annotation files reflect our current knowledge about a given genome, they are not strictly standardized. As a consequence, elements can be unevenly described. To solve this issue, ALFA adds virtual categories called “undescribed” at the gene and the exon levels. For instance, if ALFA is supposed to display the category distribution up to the UTR regions level but a gene is only annotated up to the exon level, the reads mapping to this exon of this gene will be categorized as “undescribed exon” because there is no way to know which of the deeper categories (“UTR” or “CDS”) it should account for. At the end of this process, a list of nucleotide counts per category-biotype pair is obtained for each sample and stored in one of the output files. Eventually, nucleotides that map to a region that corresponds to two or more distinct features from the same hierarchical level are considered ambiguous and are discarded by default. In this case, the percentage of ignored read counts is displayed by the program directly on the standard output.

### Displaying the results

The last step consists in creating plots from the file produced at the previous step. From the counts file, containing the number of nucleotides in the sample for each category-biotype annotation pair, counts are grouped by category and by biotype to produce two images. On each image, two plots are displayed on top of each other. The top plot represents the raw distribution of reads among the selected type of features (categories or biotypes). The bottom plot shows the same counts divided by the total amount of the corresponding features in the reference genome. This second plot provides information about the sample enrichment (or depletion) of categories/biotypes compared to the genomic global distribution.

## Results

### ALFA can detect inconsistencies between replicates

**Fig. 1:**
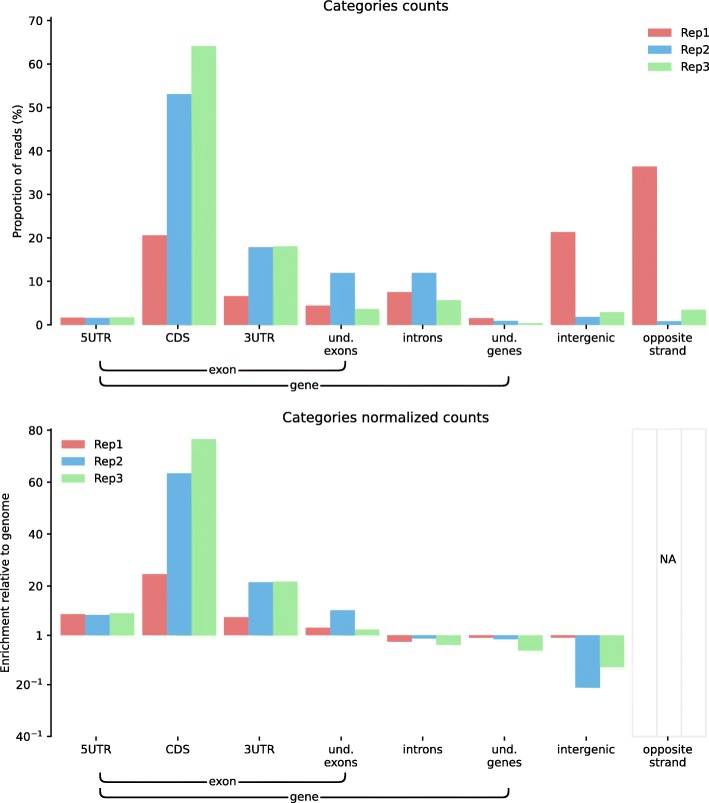
ALFA category plots (raw and normalized) for Cross-Linking and ImmunoPrecipitation Sequencing (CLIP-Seq) of eIF4A3 on *Mus musculus* in three technical replicates samples (unpublished data from HLH available on demand). Here, ALFA highlights that replicate Rep1 seems to be inconsistent with replicates Rep2 and Rep3 as CDS, 3′-UTR and intergenic categories seem to display different proportions.

Comparing feature distributions right after mapping is a simple and powerful way to easily detect possible inconsistencies between replicates. To illustrate this point, we used ALFA to evaluate the consistency of three replicates in a CLIP-Seq experiment on *Mus musculus* samples. CLIP-Seq, for Cross-Linking ImmunoPrecipitation followed by high throughput sequencing, is used to identify the binding sites of RNA associated proteins. In Fig. 1, the category plots produced by ALFA distinctly highlight that one of the replicates behaves differently from the others, with an enrichment of reads mapping within intergenic regions. In this case, ALFA allowed to quickly point out, after the mapping step, that a replicate should be further investigated, treated with special care or even discarded.

### ALFA can detect inconsistencies between experimental studies

**Fig. 2:**
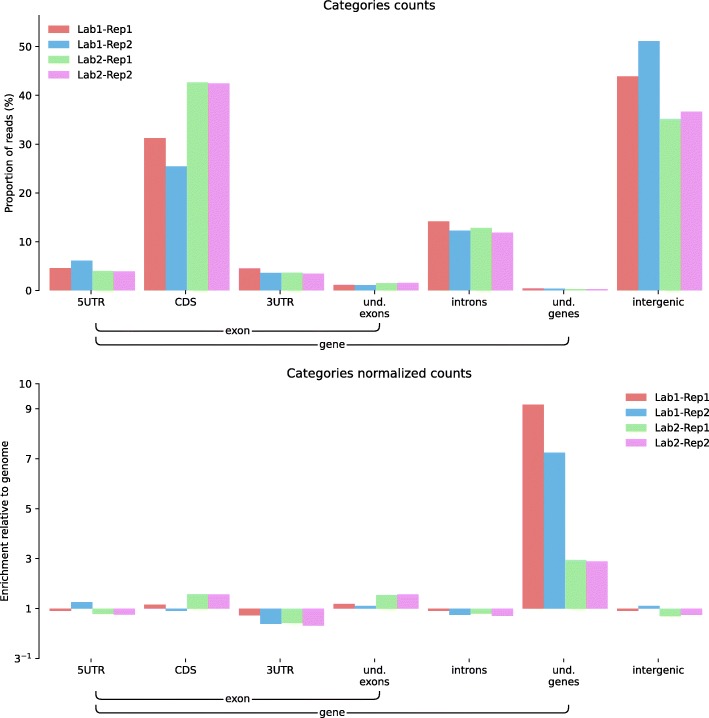
ALFA category plots (raw and normalized) for Bisulfite Sequencing (BS-Seq) data on *Arabidopsis thaliana* samples (public data available on NCBI: SRA035939 and EBI: ERA051872). Datasets were gathered from two studies performed on the same model but in two different laboratories: Lab1-Rep1 (SRR342381) and Lab1-Rep2 (SRR342391) from [14] and Lab2-Rep1 (ERR046552) and Lab2-Rep2 (ERR046553) from [15]. ALFA highlights laboratory dependent differences between reads falling in CDS (*t*-test significant at a 5% level with a *p*-value of 4 × 10^–2^).

Feature distributions can also be used to compare replicates of a similar experiment performed in different places or experimental conditions. To illustrate this point, we used ALFA to compare two BS-Seq experiments performed on *Arabidopsis thaliana* by two different laboratories ([14, 15]). BS-Seq for Bisulfite treatment followed by high throughput sequencing is used to determine the pattern of methylation on DNA. In Fig. 2, a category plot produced by ALFA permits the detection of discrepancies in the relative amount of reads mapping within coding sequences (CDS) and intergenic regions; indeed, the proportion of reads mapping to intergenic regions was higher in the data from [14] compared to [15]. Moreover, it also offered a quick overview of the reproducibility between replicates for each of these studies with [15] showing less divergence.

### ALFA can be used to validate a protocol

**Fig. 3:**
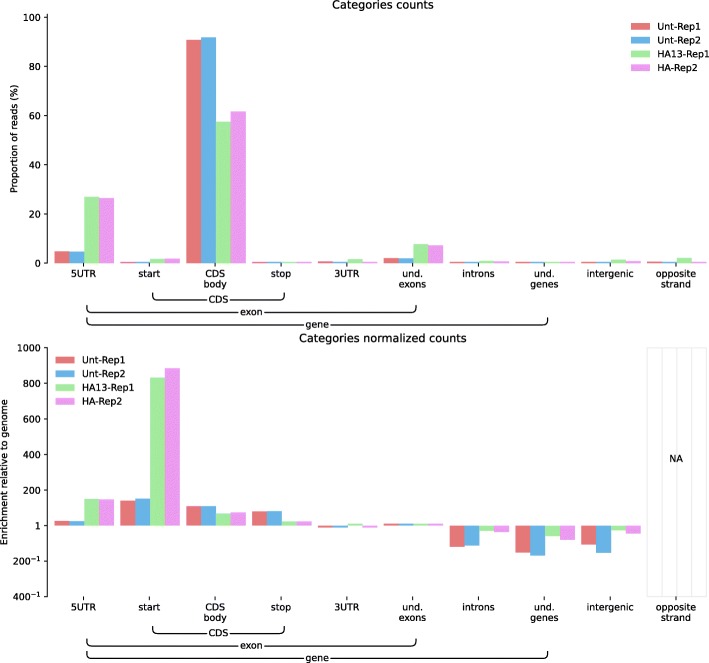
ALFA category plots (raw and normalized) for Ribosome Profiling (Ribo-Seq) data on *Mus musculus* samples (unpublished data from HLH available on demand). Unt-Rep1 and Unt-Rep2 are two untreated samples while HA-Rep1 and HA-Rep2 are samples treated with harringtonine. Harringtonine is a drug that inhibits the elongation phase of translation, after initiation. Here, ALFA shows that mRNAs are actively translated in the untreated samples (*t*-test significant at a 1% level with a *p*-value of 6 × 10^–3^) while an expected shift towards the translation start site (i.e. reads spanning the end of the 5’UTR (*p*-value = 7 × 10^–5^) and the start codons (*p*-value = 1 × 10^–3^) thanks to the depth argument set to 4) can be observed in the samples treated with harringtonine.

Feature distributions can also be used to check if a technique is working as expected. To illustrate this point we used ALFA to validate the difference between two Ribosome Profiling protocols on *Mus musculus* samples. The Ribosome Profiling technique, which consists of the deep sequencing of ribosome-protected mRNA fragments, produces a global snapshot of the translatome. Alongside two replicates using the standard protocol, we produced two extra replicates with an additional harringtonine treatment. Harringtonine is a drug that inhibits the elongation phase of translation, after initiation. In Fig. 3, the category plot produced by ALFA shows that Harringtonine decreased the CDS fraction of reads while increasing the 5’UTR and start codon fractions of reads, thus expectedly shifting the distribution of mapped reads towards the start sites.

### ALFA can help choosing between available methods

**Fig. 4:**
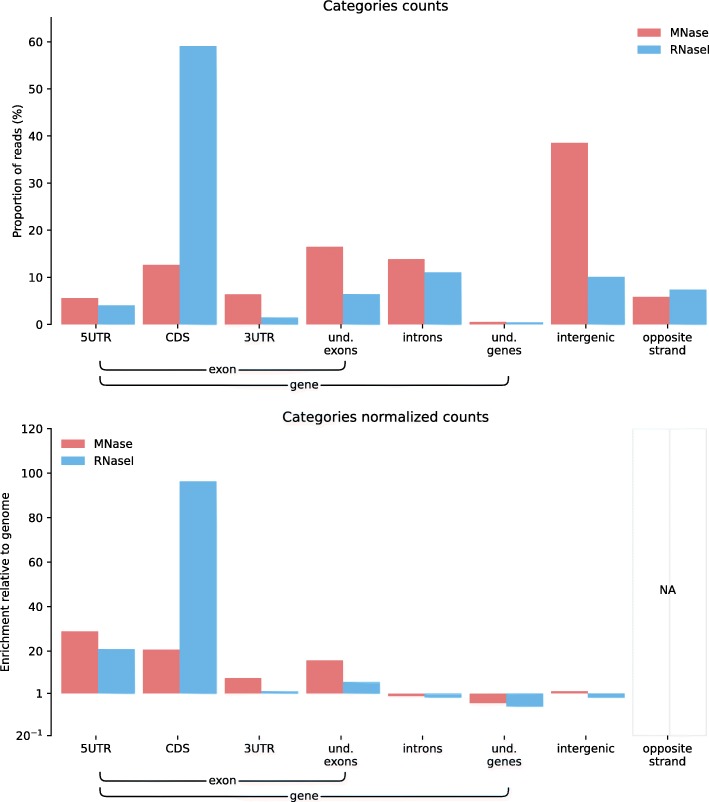
ALFA category plots (raw and normalized) for Ribosome Profiling (Ribo-Seq) data on *Homo sapiens* (unpublished data from AL available on demand) performed with two different procedures for footprinting: treatment with MNase as in [16] or treatment with RNase I as in [17]. As a preprocessing step, rRNA and mtRNA reads were computationally filtered out. The enrichment of signal in intergenic and 3′-UTR regions shows that treatment with MNase seems to produce a substantial increase of the non-protein coding reads compared to RNase I.

Comparing feature distributions can be helpful to choose a method that is adapted to the user’s experimental purpose. For instance, two Ribosome Profiling protocols have been proposed, where the footprints are generated either by digestion with MNase or with RNase I. In Fig. 4, a category plot produced with ALFA on samples treated as in [16] or as in [17] shows a substantial contamination of the footprints obtained by MNase digestion with non-protein-coding sequences, with an important fraction of reads annotated as intergenic and 3’UTR. In our hands, the MNase procedure was thus less specific of ribosome-protected RNA sequences than the RNAse I-based protocol.

### ALFA can detect contaminations

**Fig. 5:**
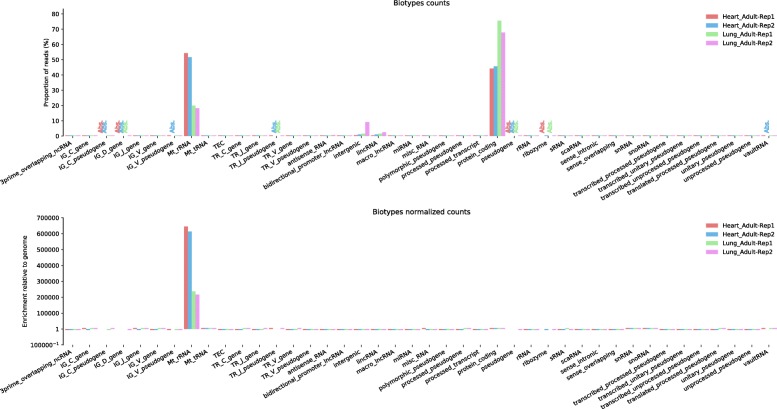
ALFA biotype plots (raw and normalized) for RNA-Seq data on *Homo sapiens* samples (public data available on NCBI: SRP058036). This dataset is part of a research work where ribosomal RNA depletion is compared between adult and fetal tissues [18]. This study reported that a large portion of transcripts with mitochondrial ribosomal origin was observed, in particular in colon, heart and kidney samples. For clarity, only lung (SRR2014234 and SRR2014235) and heart (SRR2014232 and SRR2014233) replicates from the study are reported here. ALFA enables, with a single command, to quickly confirm that mitochondrial rRNA contamination is more important in the heart samples than in the lung samples (*t*-test significant at a 1% level with a *p*-value of 3 × 10^–3^). Moreover, an intergenic contamination, not revealed in the original work, can also be noticed on Lung Adult-Rep2 with no additional work.

Comparing feature distributions enables to quickly detect a contamination. For instance, ALFA was used to identify a mitochondrial ribosomal RNA (rRNA) contamination in a RNA-Seq experiment on *Homo sapiens* samples. Contamination by rRNA can reach a few percents to the vast majority of reads depending on the experiment. In Fig. 5, a biotype plot produced by ALFA confirms a substantial rRNA contamination in one of the tissue samples as described in [18].

### ALFA provides a genomic scale overview

**Fig. 6:**
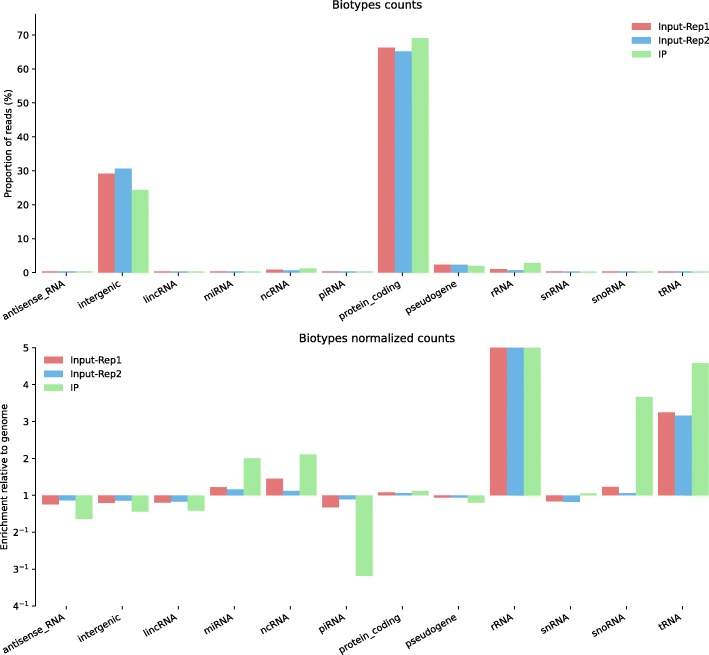
ALFA biotype plots (raw and normalized) for Chromatin Immuno-Precipitation sequencing (ChIP-Seq) of NPP-13 from *Caenorhabditis elegans* samples (public data available on NCBI: SRA062428). This dataset originates from a study [19] where snoRNA and tRNA genetic loci were found to be enriched in the IP (SRR628901) compared to the Inputs (SRR628899 and SRR628900). Here, ALFA can retrieve this result in a simple call as highlighted by this plot. Moreover, by providing a global overview without additional work, ALFA seems to denote enrichments in the IP for other biotypes such as miRNA, ncRNA genetic loci.

While primarily designed as a quality control tool, ALFA can also provide a universal way to compare samples at genomic scale prior to a dedicated analysis. For instance, the user may detect global differences between conditions as seen in Fig. 3. To further illustrate this point, we used ALFA to confirm an enrichment of specific types of genetic loci in a ChIP-Seq experiment of the nuclear pore protein NPP-13 from *Caenorhabditis elegans* performed in a previous study [19]. ChIP-Seq, for Chromatin ImmunoPrecipitation followed by high throughput sequencing, is used to identify the binding sites of DNA-associated proteins. In Fig. 6, a biotype plot by ALFA confirmed an enrichment of nuclear pore proteins at genetic loci transcribed by RNA polymerase III, such as snoRNAs and tRNAs according to the results obtained in [19]. ALFA additionally highlights that miRNA and ncRNA genes are also preferentially associated with this nuclear pore component. These loci might also correspond to polIII-transcribed genes [20], even though this would require deeper scrutiny.

### ALFA allows to use custom annotations

**Fig. 7:**
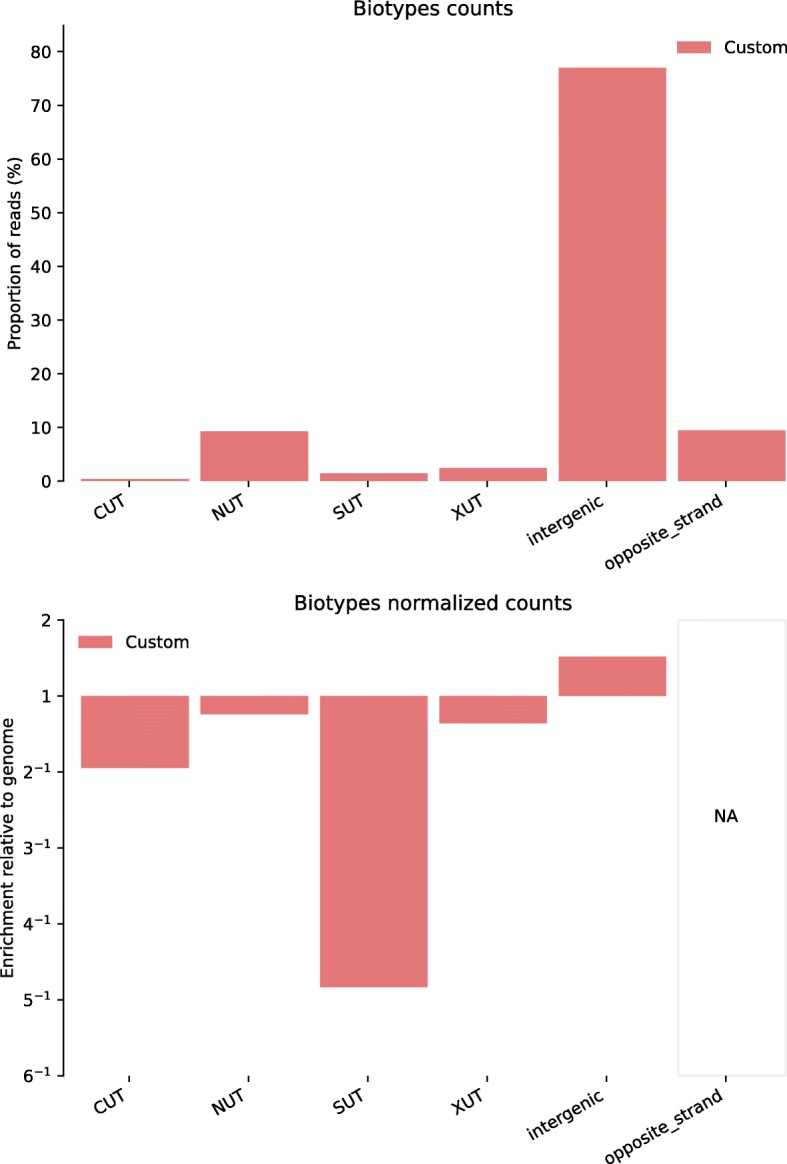
ALFA biotype plots (raw and normalized) for RNA sequencing (RNA-Seq) data from a *Saccharomyces cerevisiae* sample (public data available on NCBI: SRA030505 - SRR927165). In this example, a customized GTF annotation file was created to highlight the flexibility of ALFA. Dedicated biotypes characterizing various *Saccharomyces cerevisiae* stable transcripts (SUTs [24]) and unstable transcripts (CUTs [21], NUTs [22], XUTS [23]) were converted from a BED file.

Whereas in practice most ALFA applications should require ready-made publicly available annotation files, users may choose to define additional dedicated features of interest for specific applications. To illustrate this, we used ALFA to obtain read distributions of singular transcripts in an RNA-Seq experiment on *Saccharomyces cerevisiae*. In Fig. 7, we show that ALFA can be used with a customized genomic annotation file to observe the relative distribution of dedicated biotypes as for instance unstable (CUTs [21], NUTs [22], XUTS [23]) and stable (SUTs [24]) transcripts.

### ALFA is fast

One of the compelling advantages of ALFA, as a single integrated tool, is that it was optimized to be fast even when run on a regular desktop computer. Furthermore, it can also take advantage of multi-core machines. ALFA needs approximately 7 min to build the *Homo sapiens* genome index and 6 min to compute distributions of four samples of approximately 40 M reads using four CPUs on a regular standalone computer. Note that computing the genome index needs to be performed only once and can be reused to compute additional sample distributions.

## Discussion

In a single and simple call, ALFA proved to be useful as a post-mapping quality control tool. It was also useful to obtain a preliminary global overview of data distribution into features and functional categories prior to dedicated analysis. In this section, we emphasize the two main limitations of ALFA.

### ALFA can only detect genome scale events

ALFA is a broadly applicable and easy to use genome scale quality control tool. However, it cannot be used to perform a differential analysis at the gene level and it is not able to detect effects that would impact only a small fraction of the dataset. ALFA is meant to be a relevant post mapping quality control step but cannot replace a dedicated analysis.

### ALFA requires a GTF annotation file

ALFA cannot be used with organisms that have not been annotated yet. Therefore, the use of ALFA is somewhat restricted to organisms that are commonly studied by the scientific community and for which biotype annotations exist (e.g. GTF files from Ensembl). Alternatively, the user can provide a custom-built annotation file.

## Conclusions

In functional genomics, a vast majority of NGS experiments have the two first data analysis steps in common: a quality control of the raw sequencing reads and a mapping to the corresponding organism reference genome. We introduced ALFA, a third step that can be easily inserted into a pipeline after mapping in order to reveal genome scale possible artefacts. ALFA produces a global overview of the distribution of the mapped reads across genomic categories and biotypes at nucleotide resolution. ALFA was designed to process data from a wide variety of NGS experiments. In order to illustrate its broad usability, we applied ALFA on 6 sequencing datasets that used 5 different methods on 5 different organisms. Those examples highlighted the effectiveness of ALFA to provide a powerful post mapping quality control step on various type of NGS experiments, together with, in some cases a useful post mapping global overview. ALFA is open source and freely available as a Python script and as a Galaxy tool.

### Availability and requirements

- Project name: ALFA
- Project home page: https://github.com/biocompibens/ALFA
- Operating systems: Linux, Windows, MAC
- Programming language: Python (version 3)
- Other requirements: Python libraries: os, sys, re, numpy, collections, copy, argparse, pysam, pybedtools, matplotlib, progressbar and multiprocessing
- Licence: MIT
- Any restrictions to use by non-academics: N/A
- Availability
  - ○ GitHub: the Python code and additional information on how to use ALFA can be retrieved from the developers’ code repository at https://github.com/biocompibens/ALFA.
  - ○ PyPI: ALFA can be installed by typing the command “pip install ALFA” which downloads and sets up the script ALFA.py along with all required dependencies.
  - ○ Conda: ALFA can be installed using the command “conda install -c biocomp alfa” which also provides alfa as a standalone executable directly available from the command prompt
  - ○ Galaxy: ALFA can also be installed on Galaxy easily by just searching for “ALFA” in the main Galaxy Tool Shed (https://toolshed.g2.bx.psu.edu/).

### Reproducibility

All figures can be recomputed with the data specified in the “Availability of data and material” section below and the following ALFA command lines (optionally using the desired number of CPUs with the “-p” argument):

- Figure 1: alfa -a Mus_musculus.GRCm38 .91. chr.gtf -g M_musculus --bam Rep1.bam Rep1 Rep2.bam Rep2 Rep3.bam Rep3 -s forward
- Figure 2: alfa -a Arabidopsis_thaliana.TAIR10 .38. gtf -g A_thaliana --bam SRR342381.bam Lab1 -Rep1 SRR342391.bam Lab1 -Rep2 ERR046552.bam Lab2 -Rep1 ERR046553.bam Lab2 -Rep2
- Figure 3: alfa -a Mus_musculus.GRCm38 .91. chr.gtf -g M_musculus --bam HA -Rep1.bam HA -Rep1 HA-Rep2.bam HA-Rep2 Unt -Rep1.bam Unt -Rep1 Unt -Rep2.bam Unt -Rep2 -s forward --categories_depth 4
- Figure 4: alfa -a Homo_sapiens.GRCh38 .91. chr.gtf -g H_sapiens --bam MNase.bam MNase RNaseI.bam RNaseI
- Figure 5: alfa -a Homo_sapiens.GRCh38 .91. chr.gtf -g H_sapiens --bam SRR2014232.bam Heart_Adult -Rep1 SRR2014233.bam Heart_Adult -Rep2 SRR2014234.bam Lung_Adult -Rep1 SRR2014235.bam Lung_Adult -Rep2
- Figure 6: alfa -a Caenorhabditis_elegans.WBcel235 .91. gtf -g C_elegans --bam Input1.bam Input1 Input2.bam Input2 IP.am IP -t 5 5
- Figure 7: alfa -a Saccharomyces_all_UTs.gtf -g S_cerevisiae_custom_ -UTs_annot --bam SRR927165.bam Custom -s reverse

## Abbreviations

BAM: Binary Alignment Map
BED: Browser Extensible Data
BS-Seq: Bisulfite Sequencing
CDS: CoDing Sequences
ChIP-Seq: Chromatin ImmunoPrecipitation Sequencing
CLIP-Seq: Cross-Linking ImmunopPrecipitation Sequencing
CPU: Central Processing Unit
CUT: Cryptic Unstable Transcripts
GTF: General Transfer Format
IG-gene: ImmunoGlobulins-gene
miRNA: microRNA
MNase: Micrococcal Nuclease
mRNA: messenger RNA
mtRNA: mitochondrial RNA
ncRNA: non-coding RNA
NGS: Next-Generation Sequencing
NPP-13: Nuclear Pore Protein 13
NUT: Nrd1-Unterminated Transcripts
PyPI: Python Package Index
Ribo-Seq: Ribosome profiling
RNase: RiboNuclease
RNA-Seq: RNA Sequencing
rRNA: ribosomal RNA
snoRNA: Small Nucleolar RNA
SUT: Stable Unannotated Transcripts
tRNA: Transfer RNA
UTR: Untranslated region
XUT: Xrn1-sensitive Unstable Transcripts

## References

[1] FastQC. http://www.bioinformatics.babraham.ac.uk/projects/fastqc.

[2] Dobin A, Davis CA, Schlesinger F, Drenkow J, Zaleski C, Jha S, Batut P, Chaisson M, Gingeras TR (2013). STAR: ultrafast universal RNA-seq aligner. Bioinformatics.

[3] Barski A, Cuddapah S, Cui K, Roh TY, Schones DE, Wang Z, Wei G, Chepelev I, Zhao K (2007). High-resolution profiling of histone methylations in the human genome. Cell.

[4] Yeo GW, Coufal NG, Liang TY, Peng GE, Fu XD, Gage FH (2009). An RNA code for the FOX2 splicing regulator revealed by mapping RNA-protein interactions in stem cells. Nat Struct Mol Biol.

[5] Hower V, Starfield R, Roberts A, Pachter L (2012). Quantifying uniformity of mapped reads. Bioinformatics.

[6] Calviello L, Mukherjee N, Wyler E, Zauber H, Hirsekorn A, Selbach M, Landthaler M, Obermayer B, Ohler U (2016). Detecting actively translated open reading frames in ribosome profiling data. Nat Methods.

[7] Shin H, Liu T, Manrai AK, Liu XS (2009). CEAS: cis-regulatory element annotation system. Bioinformatics.

[8] Sun Z, Evans J, Bhagwate A, Middha S, Bockol M, Yan H, Kocher JP (2014). CAP-miRSeq: a comprehensive analysis pipeline for microRNA sequencing data. BMC Genomics.

[9] Heinz S, Benner C, Spann N, Bertolino E, Lin YC, Laslo P, Cheng JX, Murre C, Singh H, Glass CK (2010). Simple combinations of lineage-determining transcription factors prime cis-regulatory elements required for macrophage and B cell identities. Mol Cell.

[10] Quinlan AR, Hall IM (2010). BEDTools: a flexible suite of utilities for comparing genomic features. Bioinformatics.

[11] Neph S, Kuehn MS, Reynolds AP, Haugen E, Thurman RE, Johnson AK, Rynes E, Maurano MT, Vierstra J, Thomas S, Sandstrom R, Humbert R, Stamatoyannopoulos JA (2012). BEDOPS: high-performance genomic feature operations. Bioinformatics.

[12] bam2x. https://github.com/nimezhu/bam2x.

[13] Jiang Z, Yang J, Dai A, Wang Y, Li W, Xie Z. Ribosome profiling reveals translational regulation of mammalian cells in response to hypoxic stress. BMC Genomics. 2017;18(638). 10.1186/s12864-017-3996-8 PMC5563900 28826393

[14] Schmitz RJ, Schultz MD, Lewsey MG, O’Malley RC, Urich MA, Libiger O, Schork NJ, Ecker JR (2011). Transgenerational epigenetic instability is a source of novel methylation variants. Science.

[15] Becker C, Hagmann J, Muller J, Koenig D, Stegle O, Borgwardt K, Weigel D (2011). Spontaneous epigenetic variation in the Arabidopsis thaliana methylome. Nature.

[16] Reid DW, Shenolikar S, Nicchitta CV. Simple and inexpensive ribosome profiling analysis of mRNA translation. Methods. 2015. 10.1016/j.ymeth.2015.07.003 PMC4684803 26164698

[17] Ingolia NT, Ga B, Rouskin S, McGeachy AM, Weissman JS (2012). The ribosome profiling strategy for monitoring translation in vivo by deep sequencing of ribosome-protected mRNA fragments. Nat Protoc.

[18] Choy JYH, Boon PLS, Bertin N, Fullwood MJ: A resource of ribosomal RNA-depleted RNA-Seq data from different normal adult and fetal human tissues. Scientific data 2015, 2:150063, [http://www.ncbi.nlm.nih.gov/pubmed/26594381, http://www.pubmedcentral.nih.gov/articlerender.fcgi?artid=PMC4640133]. 10.1038/sdata.2015.63 PMC4640133 26594381

[19] Ikegami K, Lieb JD (2013). Integral nuclear pore proteins bind to pol III-transcribed genes and are required for pol III transcript processing in C. Elegans. Mol Cell.

[20] Dieci G, Fiorino G, Castelnuovo M, Teichmann M, Pagano A (2007). The expanding RNA polymerase III transcriptome. Trends Genet.

[21] Thompson DM, Parker R (2007). Cytoplasmic decay of intergenic transcripts in Saccharomyces cerevisiae. Mol Cell Biol.

[22] Schulz D, Schwalb B, Kiesel A, Baejen C, Torkler P, Gagneur J, Soeding J, Cramer P (2013). Transcriptome surveillance by selective termination of noncoding RNA synthesis. Cell.

[23] van Dijk EL, Chen CL, d’Aubenton Carafa Y, Gourvennec S, Kwapisz M, Roche V, Bertrand C, Silvain M, Legoix-Ne P, Loeillet S, Nicolas A, Thermes C, Morillon A (2011). XUTs are a class of Xrn1-sensitive antisense regulatory non-coding RNA in yeast. Nature.

[24] Xu Z, Wei W, Gagneur J, Perocchi F, Clauder-Munster S, Camblong J, Guffanti E, Stutz F, Huber W, Steinmetz LM (2009). Bidirectional promoters generate pervasive transcription in yeast. Nature.

[25] Liao Y, Smyth GK, Shi W (2014). featureCounts: an efficient general purpose program for assigning sequence reads to genomic features. Bioinformatics.

[26] Wang L, Wang S, Li W (2012). RSeQC: quality control of RNA-seq experiments. Bioinformatics.

